# Objectively Measured Sedentary Behavior and Physical Fitness in Adults: A Systematic Review and Meta-Analysis

**DOI:** 10.3390/ijerph17228660

**Published:** 2020-11-21

**Authors:** Fernanda M. Silva, Pedro Duarte-Mendes, Marcio Cascante Rusenhack, Meirielly Furmann, Paulo Renato Nobre, Miguel Ângelo Fachada, Carlos M. Soares, Ana Teixeira, José Pedro Ferreira

**Affiliations:** 1 Research Unit for Sport and Physical Activity (CIDAF, UID/PTD/04213/2019), University of Coimbra, 3040-248 Coimbra, Portugal; marciocascante@gmail.com (M.C.R.); prnobre@fcdef.uc.pt (P.R.N.); miguelfachada@fcdef.uc.pt (M.Â.F.); carlos.soares@student.fcdef.uc.pt (C.M.S.); ateixeira@fcdef.uc.pt (A.T.); jpferreira@fcdef.uc.pt (J.P.F.); 2 Department of Sports and Well-being, Polytechnic Institute of Castelo Branco, 6000-266 Castelo Branco, Portugal; pedromendes@ipcb.pt; 3 Sport, Health & Exercise Research Unit (SHERU), Polytechnic Institute of Castelo Branco, 6000-266 Castelo Branco, Portugal; 4 School of Physical Education and Sports, University of Costa Rica, San José 11501-2060, Costa Rica; 5 Interdisciplinary Graduate Program in Community Development, State University of the Midwest, Irati Paraná 84505-677, Brazil; meiry.furmann2@gmail.com

**Keywords:** sedentary time, accelerometry, physical capability, performance, cardiorespiratory fitness, strength, adults, meta-analysis

## Abstract

*Background*: Sedentary behavior has been considered an independent risk factor to health. The aim of this systematic review and meta-analysis was to examine associations between objectively measured sedentary time and physical fitness components in healthy adults. *Methods*: Four electronic databases (Web of Science, Scopus, Pubmed and Sport Discus) were searched (up to 20 September 2020) to retrieve studies on healthy adults which used observational, cohort and cross-sectional designs. Studies were included if sedentary time was measured objectively and examined associations with the health- or skill-related attributes of physical fitness (e.g., muscular strength, cardiorespiratory fitness, balance). After applying additional search criteria, 21 papers (11,101 participants) were selected from an initial pool of 5192 identified papers. *Results*: Significant negative associations were found between total sedentary time with cardiorespiratory fitness (r = −0.164, 95%CI: −0.240, −0.086, *p* < 0.001), muscular strength (r = −0.147, 95%CI: −0.266, −0.024, *p* = 0.020) and balance (r = −0.133, 95%CI: −0.255, −0.006, *p* = 0.040). *Conclusions*: The evidence found suggests that sedentary time can be associated with poor physical fitness in adults (i.e., muscular strength, cardiorespiratory fitness and balance), so strategies should be created to encourage behavioral changes.

## 1. Introduction

Physical fitness can be defined as a set of health- or skill-related attributes (e.g., cardiorespiratory fitness (CRF), muscular strength, balance, flexibility) that persons possess or achieve in order to carry out daily tasks [1]. Lower levels of physical fitness have been associated with a physical disability and loss of independence [2], increased falling risk and fractures [3] and increased risk of diseases with advancing age [4,5]. Furthermore, evidence shows that CRF and musculoskeletal fitness (mainly grip strength) are predictors of morbidity and all-cause mortality among middle-aged and older adults [6,7,8]. Flexibility, another health-related physical fitness component, is associated with better functional state, better ability to perform daily living activities [9] and better quality of life [10]. However, and unlike muscular strength and CRF, flexibility does not predict all-cause mortality [11]. Balance, as a skill-related component, has also been positively associated with functional capacity and can be predictive of physical independence in daily activities with age [12]. Thus, preservation of physical fitness has become a major public health concern, where the potential role of physical activity (PA) in maintaining functional independence and disease prevention has been recognized by many health organizations [4]. In turn, despite several studies supporting the positive association between PA and several health indicators (e.g., physical fitness), sedentary behavior (SB) is considered a new risk factor to health among adults, even after adjusting for PA levels [13,14,15]. SB, which is distinct from physical inactivity, is defined by any waking behavior characterized by an energy expenditure of ≤1.5 metabolic equivalents (METs), while in a sitting, reclining or lying posture [16]. A recent study revealed that adults (aged between 20 and 75 years) of four different European countries presented an average sedentary time (ST) of 8.83 h per day [17].

Available data suggest that ST is detrimental to cardiometabolic health [18] and is associated with increased risk of all-cause mortality in adults [19,20]. Regarding adults’ physical fitness, evidence shows that larger amounts of ST are associated with lower muscular strength [21,22,23,24], lower CRF [25,26,27,28] and lower balance [21,29]. However, other studies demonstrated that ST was not significantly associated with impaired muscular strength [30,31,32] and CRF [33,34,35] in adults. Even though some studies have focused on this topic, the associations between total ST and physical fitness components are still unclear. Moreover, it should be noted that a lot of studies employ subjective measures of ST, which are known to reduce data accuracy significantly, because of misreporting and recall bias [18,36]. Furthermore, cultural and linguistic issues in the interpretation of questions and/or concepts used could make comparability between countries and cultures difficult [17]. Thus, it is advisable to use objective instruments to assess ST, in order to avoid subjective biases [36]. Regarding physical fitness components, Campbell et al. [37] conducted a recent systematic review and meta-analysis to analyze the associations between ST with body weight and composition in adults and concluded that there were no significant associations between SB and any measure of body weight or obesity, with the exception of waist circumference. Thus, body composition (as a component of physical fitness) was not included in our research strategy.

Since adults are typically physically inactive [38] and present high levels of SB (in Europe) [17], it is necessary to have a better understanding of the associations between ST and physical fitness. If such detrimental associations are verified, reducing SB can be important in preventing functional limitations and the loss of independence with age [24,39]. To the best of our knowledge, there is currently no systematic review and meta-analysis available that investigates the evidence regarding the associations between objectively measured ST and physical fitness (e.g., CRF, muscular strength, balance, speed, flexibility) in adults. Thus, the aim of the present meta-analysis is to assess the magnitude of the effects of a hypothetical association between objectively measured ST and physical fitness components in healthy adults (≥18 years old). We hypothesized that ST is negatively associated with physical fitness components in adults.

## 2. Materials and Methods

The guidelines were followed from the original checklist of the Preferred Reporting Items for Systematic Reviews and Meta-Analyses (PRISMA) [40]. The protocol for this systematic review and meta-analysis has been registered at the PROSPERO International Prospective Register of Systematic Reviews, registration number 2020: CRD42020210115.

### 2.1. Data Sources

The search was conducted in the following databases: Pubmed, Sport Discus, Web of Science and Scopus, with access made between 20th August 2020 to 20th September 2020, using the advanced meta-search option in which original articles of epidemiological studies of cross-sectional, observational, cohort and/or population-based published between 2010–2020 were included. The search strategy combined Key Medical Subject Heading (MeSH) and search indexed descriptors to refine the data: (“sedentary time” OR “sedentary behavio$r” [MeSH Terms] OR “sitting time” OR acceleromet* [MeSH Terms]) AND (“physical fitness” [MeSH Terms] OR “physical function” OR “muscle strength” [MeSH Terms] OR “muscle endurance” OR “muscle power” OR cardiorespiratory [MeSH Terms] OR “aerobic capacity” OR flexibility OR balance OR agility OR coordination OR speed OR “reaction time” [MeSH Terms]) AND (adult* [MeSH Terms] OR “healthy adult*” OR “middle age” [MeSH Terms]).

### 2.2. Eligibility Criteria and Selection of Studies

To be included in this review, studies had to meet the following criteria: (1) studies that investigated associations between objectively measured sedentary time (ST) and other variables (e.g., biochemical) but which included at least one measure of the health- or skill-related attributes of physical fitness (e.g., muscular strength, cardiorespiratory fitness, flexibility, balance, speed); (2) studies published in English-language peer-reviewed journals; (3) epidemiological studies of cross-sectional, observational, cohort and population-based designs; (4) studies that assessed the components of physical fitness (i.e., CRF, muscular strength, muscular endurance, flexibility, agility, balance, coordination, speed, power and reaction time) through validated exercise tests (not reported); (5) included habitual daily/weekly total ST measured objectively (e.g., using wearable monitors/accelerometers but not direct observation); (6) studies that reported data on adults ≥18 years of age; (7) studies on healthy adults in the community who were not recruited based on any pre-existing disease and/or conditions (e.g., cancer, type II diabetes, stroke, disability besides severe obesity, post-cardiac rehabilitation), as per the included articles’ inclusion criteria; non-pregnant and non-institutionalized populations; (8) studies published from January 2010 to September 2020.

Studies investigating these associations in adults with specific pathologies or defining ST based on noncompliance with physical activity recommendations for health [41] were not included. Likewise, studies only assessing specific periods of SB, such as during work, were excluded. All studies that did not meet the initial selection criteria, as well as those which did not report results adequately (i.e., values of correlation, sample size and *p*-value) or if the respective authors did not reply to our inquiries sent by email were excluded. Finally, articles presented in the form of congress abstracts, letters to the editor, systematic reviews, study protocols, book chapters and interventional studies were excluded.

### 2.3. Data Extraction

The studies were imported into the software EndNote X8 (Thompson Reuters, San Francisco, CA, USA) and duplicates were removed. The selection procedure of the studies was performed in phases. In the initial phase, research on potentially relevant studies was performed by two independent reviewers (F.M.S., J.P.F) based on the title and abstract. If there was doubt about the inclusion of the articles, these were selected for the next evaluation phase. In the second phase, the studies selected in the previous phases were reviewed in their entirety by two independent reviewers (F.M.S., J.P.F), taking into account the specific eligibility criteria. In case of a disagreement over the inclusion of articles, these were resolved through mediation by a third reviewer (P.D.-M). The two reviewers involved in the selection of the studies participated independently in extracting the data from the selected studies and the characteristics of each study were recorded, including the author’s name, the country where the study was carried out, sample size and their average age, methodological design, sedentary behaviour (SB) assessment (data reduction and quantification of ST), physical fitness components evaluated, central outcomes, main goal and conclusions. In this phase, the discrepancies about the extracted data were resolved by consensus among the reviewers.

### 2.4. Quality Assessment

The guidelines were followed from the original PRISMA checklist [40] which describes the four stages (identification, screening, eligibility and final selection) needed to achieve the search and selection of papers and also featured the graphic option to draw a flowchart. PRISMA presents the PICOS acronym (“patient, problem or population”, “intervention”, “comparison, control or comparison”, “outcomes” and “study design”), which contributed to our research question and our systematic research being more effective [42]. To evaluate the quality of the studies, we choose to use the Strengthening Reporting of Observational Studies in Epidemiology (STROBE) statement [43,44], which consists of a checklist of 22 items. If the studies met all the criteria listed, we would give a total score of 22, which would be 100%. This procedure would not be intended as a condition for the study to be included in our meta-analysis, but rather to identify studies in which poor-quality assessment could interfere with outcomes. One researcher (F.M.S.) conducted the quality scoring, which was thereafter re-examined by one of the co-authors (J.P.F.).

### 2.5. Statistical Analysis

Meta-analysis was conducted when at least three studies were investigating the same exposure (total ST) and outcome (e.g., CRF, muscular strength, flexibility), using the same design (e.g., cross-sectional) and reporting correlation values, *p*-values and sample sizes. When the correlation value was not available from the unadjusted models, the value of the model with the smallest number of additional covariates was used. The studies included in the meta-analysis were combined using the random-effects model, including the assumption of heterogeneity of the studies and their participants [45,46]. Heterogeneity was assessed using chi-square, Cochran’s Q statistic, Higgin I squared (*I*^2^) and Tau square tests (*T*^2^). We used the Cochran’s Q test providing the possibility to test the null hypothesis that all studies in the analysis share a common effect size [47]. If all studies shared the same effect size, the expected Q value would be equal to the number of degrees of freedom (i.e., the number of studies minus 1) [47]. The *I*^2^ test represents the proportion (percentage) of the variance attributed to the heterogeneity of the study; values of 25%, 50% and 75% were considered to indicate low, moderate and high heterogeneity, respectively [47,48]. The *T*^2^ is the variance of the true effect sizes (in log units) among studies [47], assuming that *T*^2^ > 1 suggests the presence of substantial heterogeneity [48]. To evaluate the risk of publication bias, a funnel plot and Egger’s intercept test were used [48]. The capacity of this test to detect bias is limited when meta-analysis is based on a small number of studies [48]. Thus, this test is performed when there are at least ten studies included in a meta-analysis [45]. All meta-analysis was performed using Comprehensive Meta-Analysis (CMA) (version 3.0, Biostat Inc., Englewood, NJ, USA). The statistical significance of the results was set at *p* < 0.05.

## 3. Results

### 3.1. Data Search

**Figure 1:**
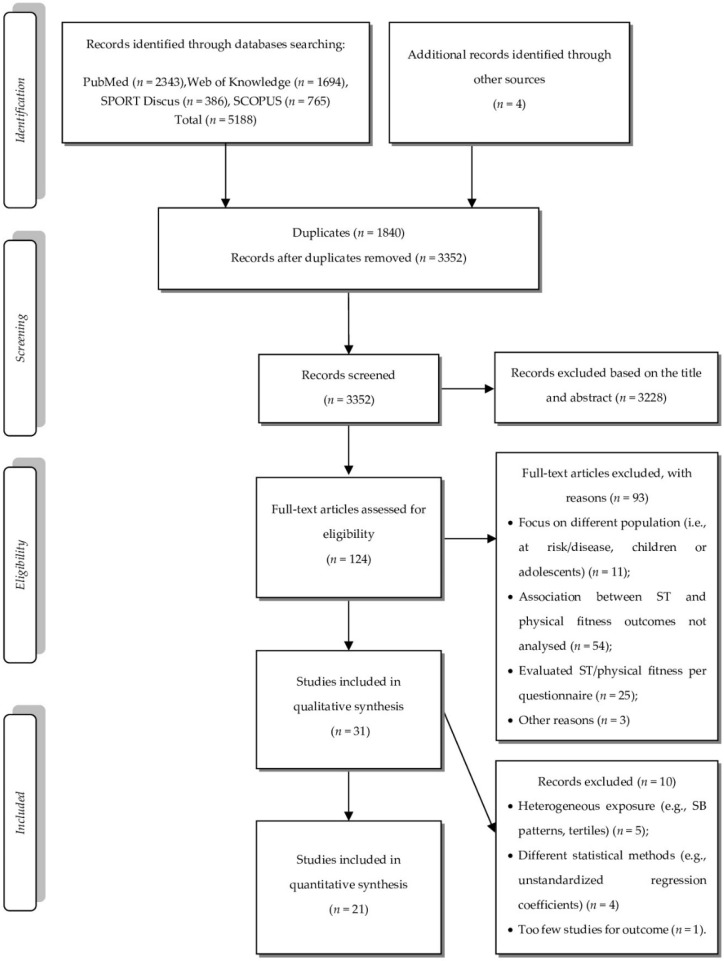
Flowchart illustrating each phase of the search and selecting process.

The sequence followed for the selection of final studies that were later included in the meta-analysis is shown in Figure 1. The initial search identified 5192 potentially eligible studies. After excluding studies following a review of titles and abstracts, duplicates and other factors, 124 full-text articles were examined in the eligibility stage. From the eligibility phase, criteria such as the use of questionnaires to measure ST and physical fitness components, non-association between ST and physical fitness, or selection of participants based on pre-existing diseases and/or conditions were the most common reasons for excluding articles. In total, 31 studies were included in the qualitative synthesis. Of these, 10 studies were excluded for the reasons presented in Figure 1, resulting in 21 studies eligible for quantitative synthesis.

### 3.2. Characteristics of Studies and Participants

Table 1 shows the 21 studies included for quantitative data meta-analysis, as well as the results of the quality assessment. Burzynska et al. [49], Dogra et al. [22] and Jantunen et al. [50] were the studies that had the lowest quality score (18 points, which corresponds to 82%). Only one study [29] had 22 points, corresponding to 100%. The studies were, in general, of high quality according to the STROBE checklist (the mean of the total scores was 90%).

Details of the 21 studies included in the systematic review and assessed for quantitative analysis are presented in Table 2. These studies included a total sample of 11,101 participants. Among them, five studies were conducted in the United States, two were in Australia, two were in Canada, two were in the United Kingdom, two were in Japan, two were in South Africa and the other six studies were performed in Europe (including Finland, Belgium, Portugal). All participants were older than 18 years old. The ages of the participants ranged from 19.0 to 78.1. A total of 18 studies presented samples from male and female participants, but only the studies carried out by Wientzek et al. [26], Gennuso et al. [51] and Spartano et al. [23] presented a separate analysis performed by gender. Studies from Willoughby and Copeland [29], Wu et al. [30] and Dickie et al. [25] include only female participants. All studies included had a cross-sectional design.

The studies used different instruments to measure ST, namely Actigraph accelerometers (GT1M, GT3x, MTI 7164 models), ActiHeart CamNtech, Actical monitors, activPAL monitors, SenseWear Pro 3 Armband, Active Style Pro HJA-350IT and UKK RM42 accelerometers. The assessment of components of physical fitness was also different between the studies. CRF was evaluated in 13 studies [22,25,26,27,33,34,35,49,50,51,53,54,55]. Muscular strength was also assessed in several studies, with tests to assess upper and lower limb strength [13,21,22,23,29,30,31,50,51,52,53,54,56]. The flexibility of the lower and upper limbs was evaluated in four studies [22,50,53,54]. Lastly, a balance component was assessed in five studies [13,21,29,30,56]. In the included studies, the commonly used modes to assess CRF included treadmills and cycle ergometers (e.g., Submaximal Treadmill Test) [27,34,35,49], steps (e.g., Submaximal Ramped Step Test) [22,25,26,33] and field tests (e.g., 6MWT) [50,51,53,54,55]. To assess muscular strength, studies commonly used the handgrip strength test to assess upper limb strength [21,22,23,52,56] and knee extension, leg strength [29,31] and chair stand [21,23,50,51,53,54] to assess lower limb strength. Regarding flexibility, the tests commonly used in the included studies were chair sit-and-reach and back scratch [22,50,53,54]. The balance component was also assessed using different tests, such as standing balance time [21] and one-legged stance with eyes open [56]) (Table 2).

### 3.3. Synthesis of Quantitative Data

The analyses were performed for each component of physical fitness (i.e., muscular strength, cardiorespiratory fitness, flexibility and balance). Importantly, the correlations reported were not adjusted for PA levels.

#### 3.3.1. Cardiorespiratory Fitness

**Figure 2:**
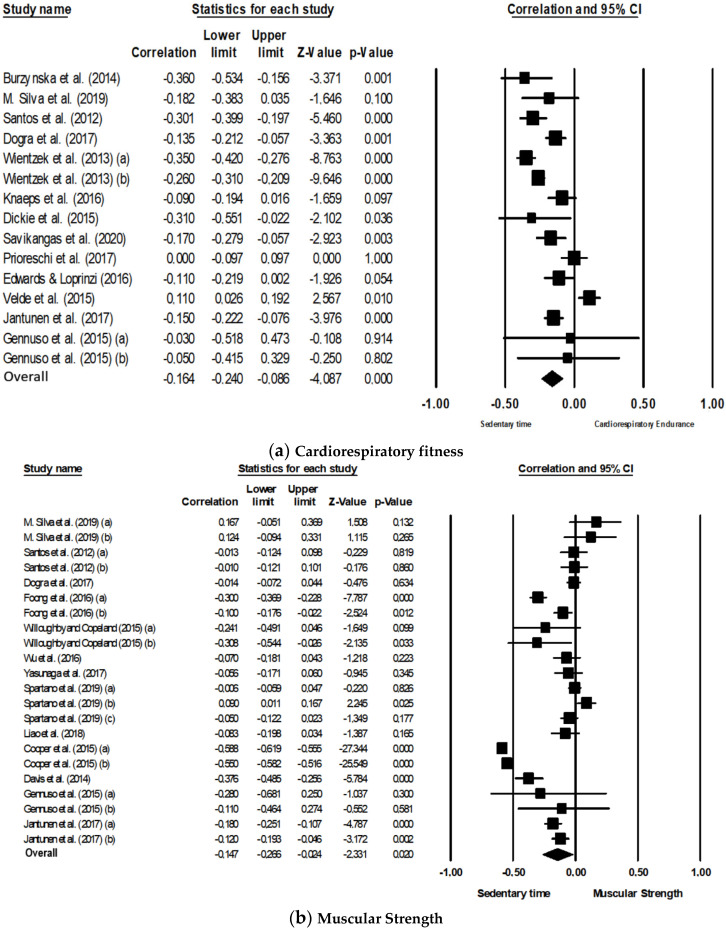

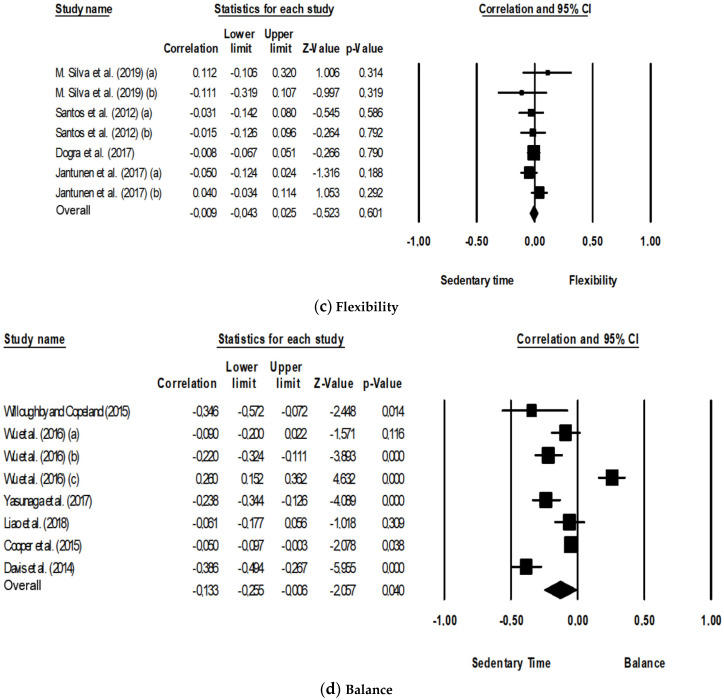
Forest plot for the association between objectively measured total ST and physical fitness components; (**a**) cardiorespiratory fitness; (**b**) muscular strength; (**c**) flexibility; (**d**) balance.

**Figure 3:**
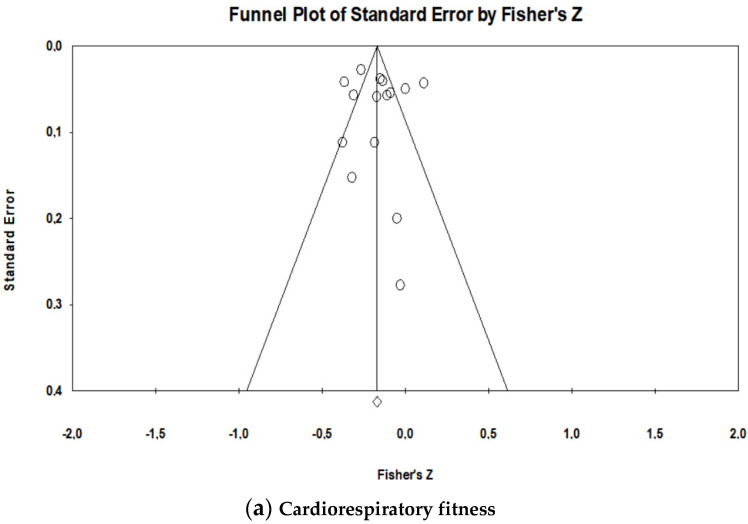

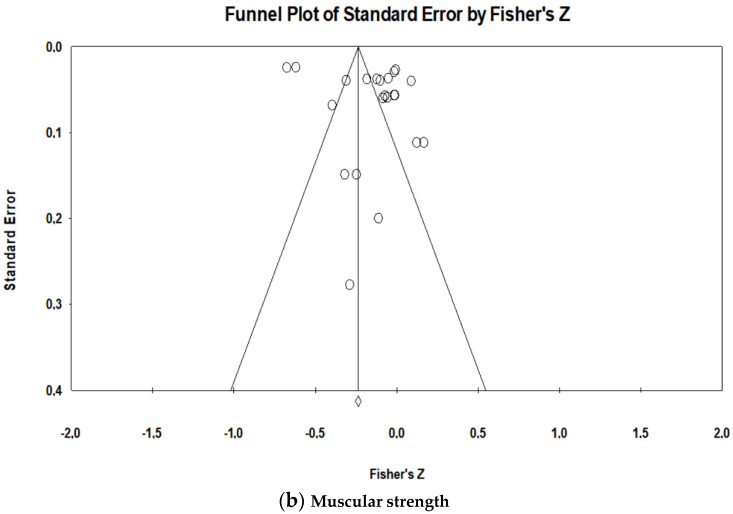
Visual funnel plot inspection associated with Egger’s test for the association between objectively measured total ST and physical fitness components; (**a**) cardiorespiratory fitness; (**b**) muscular strength.

The meta-analysis examining the cross-sectional association between overall ST and cardiorespiratory fitness included 13 studies. The number of entries was higher because the authors such as Wientzek et al. [26] and Gennuso et al. [51] presented the results separately by gender (15 entries in total). A random effects model was used to run the meta-analysis and the results showed a statistically significant negative association (r = −0.164, 95% CI: −0.240, −0.086, *p* < 0.001) between overall ST and cardiorespiratory fitness (Figure 2a). The Z-value for testing the null hypothesis was −4.087 (*p* < 0.001), thus we may reject the null hypothesis that overall ST had no association with cardiorespiratory fitness. Regarding the homogeneity of the effects, the value of the Q test was 103.64, with 14 degrees of freedom and *p* < 0.001, so we can accept the alternative hypothesis, i.e., the true magnitude of the effect size varies from study to study. The *I*^2^ was 86.491, which means that about 86.49% of the variance of the observed effects reflects the variance of the true effects. The value of *T^2^* in this same analysis was 0.018. To assess publication bias, we performed a visual inspection of the funnel plot, suggesting low evidence for publication bias (Figure 3a). In addition, the Egger’s test was performed, which proposes to test the null hypothesis according to which the intercept is equal to zero. In the analyses of ST with cardiorespiratory fitness the intercept result was 0.43570 (SE = 1.58, IC: 95% = −2.99, 3.865, t = 0.27, df = 13, *p* = 0.788). The *p*-value was not significant, indicating no strong evidence for publication bias.

#### 3.3.2. Muscular Strength

The meta-analysis examined the cross-sectional association between overall ST and muscular strength from 13 studies. The number of entries was higher because the authors used more than one measurement of muscular strength. Authors such as Silva et al. [53], Santos et al. [54] and Jantunen et al. [50] assessed upper and lower limb strength; Foong et al. [31] assessed leg strength and knee extension strength; Willoughby and Copeland [29] assessed relative peak torque extensors/flexors; Spartano et al. [23] assessed lower limb strength and handgrip strength (the last being separated by gender) and Cooper et al. [21] also evaluated lower limb strength and handgrip strength (22 entries). Results showed that the global association between ST and muscular strength was statistically significant (r = −0.147, 95% CI: −0.266, −0.024, *p* = 0.020) (Figure 2b). The Z-value for testing the null hypothesis was −2.331 with a corresponding value of *p* = 0.020. Thus, we can reject the null hypothesis that ST has no association with muscular strength. Regarding the homogeneity of the effects, the value obtained for the Q test was 896.046, with 21 degrees of freedom and *p* < 0.001, so we accept the alternative hypothesis suggesting that the true magnitude of the effect size varies from study to study. The obtained value of *I*^2^ was 97.66, so about 98% of the variance from the observed effects reflects the variance of the true effects and the value of *T^2^* was 0.081. Regarding publication bias, we performed a visual inspection of the funnel plot presented in Figure 3b, suggesting low evidence for publication bias. The intercept result was −4.45 (SE = 2.81, IC: 95% = −1.40, 10.31, t = 1.58, df = 20, *p* = 0.13), which means there is no strong evidence for publication bias.

#### 3.3.3. Flexibility

The meta-analysis examining the cross-sectional association between overall ST and flexibility included four studies, however, the authors assessed the flexibility of lower and upper limbs [50,53,54] (seven entries). The random effects model used in the meta-analysis indicated that the association between ST and flexibility was not statistically significant (r = −0.009, 95% CI: −0.043, 0.025, *p* = 0.601) (Figure 2c). The Z-value for testing the null hypothesis was −0.523 (*p* = 0.601), so we can accept the null hypothesis that ST has no associated with flexibility. The value obtained from the Q test was 5.01, with 6 degrees of freedom and *p* = 0.542, so we can accept the null hypothesis (i.e., all studies share the same magnitude of effects). Furthermore, the obtained values of *I*^2^ and *T^2^* were 0% and 0.000, respectively.

#### 3.3.4. Balance

The meta-analysis examining the cross-sectional association between overall ST and the balance component included five studies, however, Willoughby and Copeland [29] presented three different tests (seven entries). A random effects model was used to run the meta-analysis. The results showed a statistically significant negative association (r = −0.133, 95%CI: −0.255, −0.006, *p* = 0.040) (Figure 2d). The Z-value for testing the null hypothesis was −2.057 (*p* = 0.040), so we can reject the null hypothesis. The Q test value obtained was 78.21, with 7 degrees of freedom and *p* < 0.001, so we may confirm that the true magnitude of the effect size varies from study to study. The obtained value of *I*^2^ was 91.05, i.e., about 91% of the variance of the observed effects reflects the variance of the true effects. The value of *T^2^* in this analysis was 0.170.

## 4. Discussion

The present systematic review and meta-analysis aimed to examine the magnitude of the effect size between objectively measured ST and physical fitness components in healthy adults. Thus, analyses were performed for each component of physical fitness (i.e., muscular strength, cardiorespiratory fitness, flexibility and balance). Overall, 5192 records were identified and 21 articles were ultimately included. The geographic distribution and the diversity of the countries involved in the analyzed studies demonstrated that there is a large-scale concern about the impact of SB on health and well-being. Moreover, the high quality of the studies included in the present meta-analysis revealed high scientific rigor by the researchers in this specific area. In spite of the findings from individual studies seeming ambiguous, when the data were analyzed using meta-analysis we can verify that ST shows a negative association with some physical fitness components, i.e., CRF, muscular strength and balance.

CRF is a well-established predictor of several adverse health outcomes [57], morbidity and all-cause mortality [6]. Whereas factors such as age, body mass index and waist circumference show consistent evidence of an association with CRF, other factors show conflicting or insufficient evidence of such association, for example, the relationship between CRF and behavioral factors [57]. Our results suggest that total ST is negatively associated with CRF in adults and older adults. According to the results of individual studies, 8 of 13 studies included found a significant negative association [22,25,26,27,49,50,54,55], whereas 5 studies found no association [33,34,35,51,53]. These discrepancies between studies may be attributable, at least in part, to the heterogeneity of the participants from the different study samples analyzed and the characteristics of device/ data reduction procedures [18,58]. Studies carried out by Gennuso et al. [51] and Silva et al. [53] had a reduced sample size when compared with all the other studies. Knaeps et al. [34] had a heterogeneous sample with ages ranging from 29–82 years. Velde et al. [35] and Prioreschi et al. [33] had samples of young and healthy adults (ranging from 18–49 and 19–20 years old) and according to the authors’ opinion, the effects of ST possibly only become apparent in an elderly population or when moderate to vigorous PA or fitness levels decrease below a certain threshold. Furthermore, Gennuso et al. [51] was the only study included in our meta-analysis that used a posture-based activity monitor to assess sedentary behavior. This may also justify the differences. The mechanisms through which SB may affect CRF are not totally understood. This hypothetical effect could be explicated by vascular changes in response to SB [28,59]. According to Thijssen et al. [59], the pathways through which these changes arise appear to be different from pathways that are involved in vascular changes in response to PA. However, we believe it is imperative to produce further research to determine the mechanisms/molecular basis by which SB affects CRF.

Upper and lower body muscular strength is also a predictor of cardiovascular mortality and all-cause mortality in adults and old people [7,8]. Adults with lower levels of muscular strength experience more difficulties when performing their daily life activities and, as a result of that, levels of PA decrease leading to greater muscle mass loss [60]. This decreasing process may justify why adults may be more susceptible to injurious falls or adverse events [8]. Results from our meta-analysis suggest that ST is negatively associated with upper and lower body strength in adults. This negative association was found in the majority of the individual studies included [13,21,22,23,29,50,54]. The mechanisms through which SB may affect muscular strength remain uncertain. Some authors have proposed that continual underloading due to SB may affect negatively the muscle-tendon proprieties, since muscle-tendon disuse causes changes such as muscle atrophy [61]. Furthermore, long periods of SB result in low energy expenditure and may contribute to weight gain and obesity [15,62]. Evidence suggests that concurrent increases in visceral and intermuscular fat can stimulate the release of pro-inflammatory cytokines and decrease anti-inflammatory markers from adipose tissue, which can have a catabolic effect on muscle by impairing muscle protein synthesis [63]. This will affect muscle mass and strength [64,65].

Regarding balance, Breton et al. [12] show that balance is associated with functional capacity and can be predictive of physical independence in daily activities with age. The risk factors for postural instability can be explained by inactivity and aging [66]. Studies show that old women have more pronounced changes in their postural stability; however, a significant decline in the ability to maintain balance in challenging environments (e.g., eyes closed) has also been observed in women over 50 years old [67]. The results of our meta-analysis seem to confirm the evidence found in the literature since they suggest a negative association between ST and balance in adults.

Flexibility declines with age [68] and is associated with better functional state and ability to perform daily activities [9] and better quality of life [10]. However, unlike muscular strength and CRF, flexibility does not predict fall [69] and all-cause mortality [11]. Our meta-analysis does not provide evidence of a negative association between ST and flexibility. Moreover, the majority of studies involved in this meta-analysis did not find a significant negative association between ST and upper and lower limb flexibility, so the role of ST in flexibility is unclear.

Few previous reviews are comparable to this work. Mañas et al. [70] performed a systematic review and concluded that the association between objectively measured SB and physical performance in old people is strong, with just one study included not showing a significant association. Wirth et al. [71] also conducted a systematic review to investigate the associations between SB (objectively measured and self-reported) and various biomarkers in older adults. The authors found five studies evaluating the associations between SB and performance and concluded that SB was associated in an unfavorable way with performance biomarkers [71]. Wullems et al. [61] also conducted a systematic review and verified that the literature reports negative associations in SB in the elderly, such as less favorable cardiometabolic health, physical functioning, musculoskeletal health and body composition; however, the authors considered the evidence so far to be inconclusive. Overall, the results of these three reviews suggest a negative association between the ST and physical fitness components, however these reviews included only studies carried out in the elderly and with subjective measures of SB.

The results of this meta-analysis have major public health implications since the decline of analyzed physical fitness components (i.e., CRF, muscular strength and balance) are associated with physical disability and loss of independence [2], falling risk and fractures [3] and an increased risk of morbidity and all-cause mortality in adults [6,7,8]. Thus, the results of this meta-analysis should be taken into consideration. In general, authors from the included studies recommended developing strategies to reduce ST and to increase PA levels among these age groups [22,29,34,53,54,55]. In addition, supplementary analyses carried out in some studies suggested a frequent interruption of ST [13,22] and replacement of even small amounts of ST with LPA (light physical activity) or MVPA (moderate to vigorous physical activity), in order to contribute to improvements in physical fitness [56]. Biddle et al. affirms that “the best posture is the next posture” and reinforces the importance of regularly breaking up ST and replacing this with postural shifts and movement to improve health [72].

The random effects model was used to run the meta-analysis because of the assumption of heterogeneity between studies (e.g., use of different instruments and data reduction to evaluate ST; different physical fitness tests) and between their participants (e.g., age of participants, social background). The results of the present meta-analysis should be interpreted with caution because of heterogeneity in the assessment of physical fitness tests and the devices used to measure ST. Moreover, our results were derived from cross-sectional studies and thus should be interpreted accordingly. Poor physical fitness levels may be a determinant as well as a consequence of SB and they may influence the context of SB [14]. In this sense, quality evidence from studies using longitudinal and/or intervention designs are needed to examine reverse-causal relationships between SB and physical fitness in adults. Furthermore, necessary studies are those that determine the molecular basis by which SB is associated with reduced CRF, muscle strength and balance [63,73,74]. Since our meta-analysis does not take into account the mediation of levels of PA, we suggest that future reviews should analyze the association between SB and physical fitness, with the mediation of PA levels.

### Strengths and Limitations

The eligibility criteria ensured that only papers that utilized objective measures to quantify cross-sectional associations between ST and physical fitness components were included. To the best of our knowledge, this systematic review and meta-analysis is the first quantitative synthesis of associations between total ST and physical fitness. Despite the high methodological quality of the included studies, this work has several limitations. First, our research strategy was limited to studies published in the English language since 2010, which may have resulted in a linguistic or cultural bias, however, our extensive research in four databases incorporated several studies conducted in different countries. Second, as there were few studies for some outcomes, meta-analysis could not be conducted to test all components of physical fitness, such as agility, coordination, speed, power and reaction time. Third, another limitation is related to the heterogeneity of samples included in the studies, which may limit the interpretation of our results. We included seven studies in adults aged between 18 to 64 years old [21,25,26,27,30,34,35] and nine studies including participants aged ≥65 years [13,23,50,51,52,53,54,55,56]. Other studies included participants aged between 50 to 67 years old [29], 50 to 80 years old [31], 60 to 78 years old [49], 29 to 82 years old [34] and 60 to 69 years old [22]. Since these studies did not perform correlation analyses by age group, it is difficult to carry out extra comparative analyses between adults and elderly. However, the meta-analysis performed by Powell et al. [18] also included studies where the age range of participants was large (18–87 years). Fourth, objective measures also have their own challenges, as there is no consensus regarding the processing and collection of accelerometer data (e.g., definitions of non-wear time and number of valid days), which can limit the interpretation of our results [75]. There needs to be evidence to reach a consensus on SB data reduction protocols. Fifth, the statistical procedures used also have their own limitations. For example, to assess the heterogeneity we used Cochran’s Q test (based on a chi-square distribution) which is known to be poor at detecting true heterogeneity, especially when the number of studies is small. However, to address this limitation, we also performed *I*^2^ and *T*^2^ tests to analyze the heterogeneity of the studies, which, unlike Q, does not depend upon the number of studies considered. Furthermore, the funnel plot and Egger’s intercept test to evaluate the risk of publication bias were not used in the flexibility and balance analysis, because the capacity of this test to detect bias is limited in meta-analysis with small number of studies (<10 studies) [48].

## 5. Conclusions

Our findings suggest that total ST is negatively associated with physical fitness components, namely CRF, muscular strength and balance in adults (≥18 years old). However, all results were derived from cross-sectional studies and thus should be interpreted accordingly. The data presented are extremely relevant to public health and strategies should be created to encourage behavioral changes in adult populations.

## Figures and Tables

**Table 1:** Quality assessment scores of selected studies (STROBE checklist).

Study	1	2	3	4	5	6	7	8	9	10	11	12	13	14	15	16	17	18	19	20	21	22	100%	22
1. Burzynska et al. (2014) [49]	1	1	1	0	0	1	1	1	1	1	1	1	0	1	1	1	1	1	0	1	1	1	82	18
2. Cooper et al. (2015) [21]	1	1	0	1	1	0	1	1	1	1	1	1	1	1	1	1	1	1	1	1	1	1	91	20
3. Davis et al. (2014) [13]	1	1	1	1	1	0	1	1	0	1	1	1	1	1	1	1	1	1	0	1	1	1	86	19
4. Dickie et al. (2015) [25]	1	1	0	1	1	1	1	1	0	1	1	1	1	1	1	1	0	1	1	1	1	1	86	19
5. Dogra et al. (2017) [22]	1	1	0	1	1	1	1	1	0	1	1	1	1	1	1	1	0	1	1	1	1	0	82	18
6. Edwards and Loprinzi (2016) [27]	1	1	1	1	1	0	1	1	0	1	1	1	1	1	1	1	1	1	1	1	1	1	91	20
7. Foong et al. (2016) [31]	1	1	0	1	1	1	1	1	0	1	1	1	1	1	1	1	1	1	1	1	1	1	91	20
8. Gennuso et al. (2015) [51]	1	1	1	1	1	0	1	1	0	1	1	1	1	1	1	1	1	1	1	1	1	1	91	20
9. Jantunen et al. (2017) [50]	1	1	0	1	1	0	1	1	0	1	1	1	1	1	1	1	1	1	1	1	1	0	82	18
10. Knaeps et al. (2016) [34]	1	1	1	1	1	0	1	1	0	1	1	1	1	1	1	1	1	1	1	1	1	1	91	20
11. Liao et al. (2018) [52]	1	1	1	1	1	1	1	1	0	1	1	1	1	1	1	1	1	1	1	1	1	1	95	21
12. Silva et al. (2019) [53]	1	1	1	1	1	1	1	1	0	1	1	1	0	1	1	1	0	1	1	1	1	1	86	19
13. Prioreschi et al. (2017) [33]	1	1	0	1	1	1	1	1	0	1	1	1	1	1	1	1	1	1	1	1	1	1	91	20
14. Santos et al. (2012) [54]	1	1	0	1	1	1	1	1	0	1	1	1	1	1	1	1	1	1	1	1	1	1	91	20
15. Savikangas et al. (2020) [55]	1	1	1	1	1	1	1	1	0	1	1	1	1	1	1	1	1	1	1	1	1	1	95	21
16. Spartano et al. (2019) [23]	1	1	1	1	1	1	1	1	0	1	1	1	1	1	1	1	1	1	1	1	1	1	95	21
17. Velde et al. (2015) [35]	1	1	0	1	1	1	1	1	0	1	1	1	1	1	1	1	1	1	1	1	1	1	91	20
18. Wientzek et al. (2013) [26]	1	1	1	1	1	1	1	1	0	1	1	1	1	1	1	1	1	1	1	1	1	1	95	21
19. Willoughby and Copeland (2015) [29]	1	1	1	1	1	1	1	1	1	1	1	1	1	1	1	1	1	1	1	1	1	1	100	22
20. Wu et al. (2017) [30]	1	1	0	1	1	1	1	1	1	1	1	1	1	1	1	1	1	1	1	1	1	1	95	21
21. Yasunaga et al. (2017) [56]	1	1	0	1	1	1	1	1	0	1	1	1	1	1	1	1	1	1	1	1	1	1	91	20
Mean of total scores																							90	20

**Table 2:** Characteristics of the 21 selected studies for systematic review and meta-analysis.

Author, Year, Country, Study Name	Sample Size (*n* total; *n* ♂/ *n* ♀)	Age (Years ± SD; Range)	Sedentary Behavior Assessment (Data Reduction and Quantification of Sedentary Time)	Physical Fitness Assessment	Central Outcomes	Main Goal	Conclusions
1. Burzynska et al. (2014) [49] United States of America	88 (33 ♂; 55 ♀)	65 ± 4 (60–78 y)	Device: ActiGraph GT3X; SB cut-point: ≤100 counts/min; Epoch: NA; Non-wear: 30 min; Minimum wear: ≥3 d, ≥600 min/day; Average wear (days and h/day): 6.8 ± 0.8 days, 13.7 ± 1.3 h/day; Quantification of SB: Total ST (h/day).	CRF (modified Balke graded maximal exercise test (mL/kg/min)).	PA levels, ST, CRF and White Matter integrity	To examine the association of both PA and CRF with measures of white matter integrity.	CRF was negatively associated with ST (r = −0.36; *p* = 0.001)
2. Cooper et al. (2015) [21] Britain MRC NSHD	1727 (837 ♂; 890 ♀)	63.3 (60–64 y)	Device: Actiheart, CamNtech; SB cut-point: ≤1.5 METs; Epoch: 30 s; Non-wear: 60 min; Minimum wear: ≥2 days; Average wear (days and min/day): NA; Quantification of SB: Total ST (h/day).	Muscular strength (handgrip strength test (kg); chair rise time (s)); balance (standing balance time (s)).	ST, MVPA, PAEE, strength, balance, gait speed	To investigate the associations of ST, MVPA and PAEE with physical capability measures at age 60–64 years.	Greater time spent sedentary was associated with lower grip strength (kg), chair rise (stands/min) and standing balance time (s) (*p* < 0.05).
3. Davis et al. (2014) [13] United Kingdom Project OPAL (Older People and Active Living)	217 (108 ♂; 109 ♀)	78.1 ± 5.8 (NA)	Device: ActiGraph GT1M; SB cut-point: ≤100 counts/min; Epoch: 60 s; Non-wear: 100 min; Minimum wear: ≥5 days, ≥600 min/day; Average wear (days and min/day): NA, 14.4 ± 1.4 h/day; Quantification of SB: Total ST (min/h).	Balance (ability to maintain tandem, semitandem and side-by-side stands for 10s (score)); muscular strength (chair rise (score)).	ST, frequency of ST breaks and lower extremity strength	To evaluate the relationship of objectively measured ST, frequency of breaks in ST and lower extremity function	Negative association between ST with balance (r = −0.386, *p* < 0.05) and lower limb strength (r = −0.376, *p* < 0.05).
4. Dickie et al. (2015) [25] South Africa	76 (76 ♀)	34 ± 7 (25–52 y)	Device: ActiGraph MTI 7164; SB cut-point: ≤100 counts/min; Epoch: 60 s; Non-wear: 60 min; Minimum wear: ≥4 days, ≥600 min/day; Average wear (days and min/day): NA; Quantification of SB: Total ST (min/day).	CRF (Submaximal MRC Step test, predicted VO_2_max (mL/kg/min)).	PA, CRF, body composition and cardiometabolic risk factors	To examine the independent associations of PA, CRF and ST on body composition and cardiometabolic risk factors for CVD and T2D in black South African women.	CRF was negatively associated with ST (r = −0.31, *p* = 0.031).
5. Dogra et al. (2017) [22] Canada Canadian Health Measures Survey	1157 (564 ♂; 593 ♀)	64 (60–69 y)	Device: Actical accelerometer; SB cut-point: ≤100 counts/min; Epoch: 60 s; Non-wear: 60 min; Minimum wear: ≥4 days, ≥600 min/day; Average wear (days and min/d): NA, 595 min/day; Quantification of SB: Total ST (min/day), ST bouts, ST breaks.	CRF (Canadian Aerobic Fitness Test (ml/kg/min)); Flexibility (Sit-and-reach (cm)); Muscular strength (hand grip strength (kg)).	ST, ST breaks, CRF and musculoskeletal fitness	To analyze the associations between total ST and ST breaks with CRF and musculoskeletal fitness.	ST was negatively associated with CRF (r = −0.135, *p* < 0.05) and handgrip strength (r = −0.014, *p* < 0.05).
6. Edwards and Loprinzi (2016) [27] US NHANES 2003-2004	307 (54.2% ♂; 45.8% ♀)	34.3 (20–49 y)	Device: ActiGraph MTI 7164; SB cut-point: ≤99 counts/min; Epoch: 60 s; Non-wear: 60 min; Minimum wear: ≥4 days, ≥600 min/d; Average wear (days and min/day): NA; Quantification of SB: Total ST (min/day).	CRF (treadmill-based CRF component (mL/kg/min)).	MVPA, ST, CRF, metabolic syndrome	To evaluate the independent and additive associations of MVPA, SB, CRF with metabolic syndrome.	ST was not associated with CRF (r = −0.11, *p* = 0.06).
7. Foong et al. (2016) [31] Australia Tasmanian Older Adult Cohort study,	636 (313 ♂; 323 ♀)	66.0 ± 6.7 (50–80 y)	Device: ActiGraph GT1M; SB cut-point: ≤100 counts/min; Epoch: NA; Non-wear: NA; Minimum wear: ≥4 days, ≥600 min/day; Average wear (days and min/day): NA; Quantification of SB: Total ST (min/day).	Muscular strength (knee extension strength (kg); leg strength (kg)).	PA, muscle mass and lower-limb strength	To describe the relationship between accelerometer-determined PA, muscle mass and lower-limb strength in community-dwelling older adults.	ST was not associated with muscular strength (leg strength, r = −0.30, *p =* 0.162; knee extension strength, r = −0.1, *p* = 0.072).
8. Gennuso et al. (2015) [51] Wisconsin	44 (16 ♂; 28 ♀)	70 ± 8 (68–76 y)	Device: activPAL PA monitor; SB cut-point: Postural classification; Epoch: NA; Non-wear: NA; Minimum wear: ≥3 days, ≥600 min/day; Average wear (days and min/day): NA; Quantification of SB: Total ST (h/day), SB bout length, break rate.	Muscular strength (chair stand (score)); CRF (400-m walk (m/s)).	Total ST, patterns of SB, strength and aerobic fitness	To examine the relationship between various objectively measured SB variables and physical function.	Total ST was not associated with muscular strength and CRF (*p* > 0.05).
9. Jantunen et al. (2017) [50] Finland Helsinki Birth Cohort Study	695 (316 ♂; 379 ♀)	70.7 ± 2.7 (NA)	Device: SenseWear Pro 3 Armband; SB cut-point: ≤1.5 MET; Epoch: NA; Non-wear: NA; Minimum wear: ≥5 days (include 1 weekend day); Average wear (days and min/day): NA, 1436.8 ± 6.0 min/day; Quantification of SB: Total ST (h/day).	CRF (6 MWT (m)); muscular strength (chair stand and arm curl (reps)); flexibility (chair sit-and-reach and Back scratch (cm)).	PA levels, ST, physical fitness	To explore the association between objectively measured PA and physical performance in old age.	ST was negatively correlated with physical fitness components (lower limb strength, r *=* −0.18, *p <* 0.001; upper limb strength, r *=* −0.12, *p <* 0.001; and CRF, r *=* −0.15, *p <* 0.001).
10. Knaeps et al. (2016) [34] Belgium Flemish longitudinal study	341 (207 ♂; 134 ♀)	53.8 ± 8.9 (29–82 y)	Device: SenseWear Pro 3 Armband; SB cut-point: ≤1.5 MET; Epoch: NA; Non-wear: NA; Minimum wear: ≥3 days (1 weekday and both weekend days), ≥1296 min/day; Average wear (days and min/day): NA; Quantification of SB: Total ST (h/day).	CRF (Cycle Ergometer, Lode, Groningen, the Netherlands, predicted VO_2_max (mL/kg/min)).	ST, MVPA, CRF, cardiometabolic risk markers	To study the independent associations of ST, MVPA and objectively measured CRF with cardiometabolic risk markers and individual components.	ST was not associated with CRF (r = −0.09; *p =* 0.11).
11. Liao et al. (2018) [52] Japan	281 (174 ♂; 107 ♀)	74.5 ± 5.2 (65–84 y)	Device: Active Style Pro HJA-350IT; SB cut-point: ≤1.5 METs; Epoch: 60 s; Non-wear: 60 min; Minimum wear: ≥4 days (include 1 weekend day), ≥600 min/d; Average wear (days and min/day): NA, 900.9 ± 86.4 min/day; Quantification of SB: Total ST (min/day), sedentary bouts, sedentary breaks.	Muscular strength (hand grip strength test (kg)); balance (eye-open one leg standing test (s)).	ST, balance, gait speed and strength	To examine the associations between objectively measured SB and physical function among older Japanese adults.	Total ST was not associated with handgrip (r = −0.083, *p =* 0.165) and balance (r = −0.061, *p* = 0.411)
12. Silva et al. (2019) [53] Portugal	83 (27 ♂; 56 ♀)	72.14 ± 5.61 (65–87 y)	Device: ActiGraph GT1M; SB cut-point: ≤100 counts/min; Epoch: 15 s; Non-wear: 60 min; Minimum wear: ≥3 days (include 1 weekend day), ≥600 min/day; Average wear (days and min/day): NA, 782.47 ± 80.59 min/day; Quantification of SB: Total ST (min/day).	CRF (6 MWT (m)); muscular strength (Chair stand and arm curl (reps)); flexibility (chair sit-and-reach and Back scratch (cm)).	PA levels, ST, physical fitness	To examine the relationship between ST, LPA and MVPA with the elderly’s physical fitness.	ST was not significantly associated with physical fitness measures (*p* > 0.05).
13. Prioreschi et al. (2017) [33] South Africa Birth to Twenty (BT20) cohort study	409 (218 ♂; 191 ♀)	NA (19–20 y)	Device: ActiGraph GT1M; SB cut-point: ≤100 counts/min; Epoch: 5 s; Non-wear: 90 min; Minimum wear: ≥3 days, ≥500 min/day; Average wear (days and min/day): NA; Quantification of SB: Total ST (min/d).	CRF (Submaximal Ramped Step Test (mlO_2_/kg/min)).	PA levels, fitness, BMI	To describe fitness and objectively measure PA levels and patterns in adults, as well as to examine associations between PA, fitness and BMI.	ST was not associated with CRF (r = 0.00, *p* = 0.42).
14. Santos et al. (2012) [54] Portugal	312 (117 ♂; 195 ♀)	74.3 ± 6.6 (65–103 y)	Device: ActiGraph GT1M; SB cut-point: ≤100 counts/min; Epoch: 15 s; Non-wear: 60 min; Minimum wear: ≥3 days (include 1 weekend day), ≥600 min/day; Average wear (days and min/day): NA; Quantification of SB: Total ST (min/day).	CRF (6 MWT (m)); muscular strength (Chair stand and arm curl (reps)); flexibility (chair sit-and-reach and back scratch (cm)).	PA levels, ST, physical fitness	To examine the independent impact of objectively measured MVPA and ST on functional fitness.	ST was negatively associated with physical fitness components (upper limb strength, r *=* −0.013, *p* < 0.05; lower limb strength, r *=* −0.010, *p* < 0.05; CRF, r *=* −0.301, *p* < 0.05).
15. Savikangas et al. (2020) [55] Finland PASSWORD -study	293 (122 ♂; 171 ♀)	74.44 ± 3.78 (70–85 y)	Device: UKK RM42 accelerometer (UKK, Tampere, Finland); SB cut-point: bin threshold <0.0167 g; Epoch: 5 s; Non-wear: 60 min; Minimum wear: ≥3 days, ≥600 min/day; Average wear (days and h/day): 6.7 days; 14.1 h/day; Quantification of SB: Total ST (min/day).	CRF (6 MWT (m)).	PA, body composition, physical function	To investigate the associations of particular PA intensities with body composition and physical function among older adults.	ST was negatively associated with CRF (r = −0.170, *p* < 0.01).
16. Spartano et al. (2019) [23] US Framingham Offspring Study	1352 (46% ♂; 54% ♀)	68.6 ± 7.5 (NA)	Device: Actical model no. 198-0200-00; SB cut-point: ≤200 cpm; Epoch: 60 s; Non-wear: 60 min; Minimum wear: ≥4 days, ≥600 min/day; Average wear (days and min/day): NA, 749 ± 71 min/day; Quantification of SB: Total ST (%/day).	Muscular strength (handgrip strength test (kg); chair stand(s)).	PA, ST, gait speed, strength	To explore associations of PA/ST with physical performance across mid-older age in adults.	ST was associated with poorer performance on chair stand test (*p* < 0.001) and handgrip in men (*p* = 0.025).
17. Velde et al. (2015) [35] US NHANES 2003–2004	543 (297 ♂; 246 ♀)	32.19 ± 0.57 (18–49 y)	Device: ActiGraph AM-7164; SB cut-point: ≤100 counts/min; Epoch: 60 s; Non-wear: 60 min; Minimum wear: ≥1 day, ≥600 min/d; Average wear (days and min/day): NA, 851.87 ± 4.5 min/day; Quantification of SB: Total ST (min/day).	CRF (submaximal treadmill test (mL/kg/min)).	ST, PA, CRF and cardiometabolic risk factors	To examine and compare the independent associations of objectively measured ST, MVPA and fitness with cardiometabolic risk factors.	The correlation between ST and CRF was r = 0.11.
18. Wientzek et al. (2013) [26] Denmark, Greece, the Netherlands, United Kingdom, Italy, Spain, France, etc. EPIC-Europe cohort	1895 (578 ♂; 1317 ♀)	53.78 ± 9.36 (NA)	Device: Actiheart, CamNtech; SB cut-point: <0.25 m/s^2^/d; Epoch: 60 s; Non-wear: NA; Minimum wear: ≥4 days, NA; Average wear (days and min/day): NA; Quantification of SB: Total ST (%/day).	CRF (8-min submaximal ramped step test (ml/kg/min));	PA, CRF and anthropometry	To quantify the independent associations between objectively measured total PA, MVPA, ST and CRF and anthropometric markers in apparently healthy European men and women.	ST was negatively associated with CRF in men (r = −0.35, *p* < 0.01) and women (r = −0.26, *p* < 0.01).
19. Willoughby and Copeland (2015) [29] Canada	49 (49 ♀)	56.6 ± 4.1 (50–67 y)	Device: ActiGraph GT3X; SB cut-point: ≤100 counts/min; Epoch: 60 s; Non-wear: 90 min; Minimum wear: ≥4 days, ≥600 min/d; Average wear (days and min/d): NA; Quantification of SB: Total ST (%/day), number of sedentary breaks.	Balance (NeuroCom Equitest CRS+ Balance Master computerized dynamic posturography system); muscular strength (peak torque of the dominant knee extensors and flexors).	ST, lower body muscular strength and postural stability	To determine whether ST is negatively associated with laboratory-based measures of lower body muscular strength and postural stability in middle-aged women.	Balance and relative peak torque of the knee flexors were significantly associated with ST (r = −0.35, *p* = 0.01 and r = −0.31, *p* = 0.03, respectively).
20. Wu e al. (2017) [30] Australia 2000 Tasmanian Electoral Roll	309 (309 ♀)	50 ± 5 (36–57 y)	Device: ActiGraph GT1M; SB cut-point: ≤150 counts/min; Epoch: 60 s; Non-wear: NA; Minimum wear: ≥5 days, ≥600 min/day; Average wear (days and min/day): NA, 851 min/day; Quantification of SB: Total ST (min/day).	Muscular strength (lower limb muscular strength (kg)); balance (step test (steps), functional reach test (cm) and lateral reach test (cm)).	PA levels, ST, lumbar spine and femoral neck, bone mineral density, muscular strength and balance	To describe associations between objectively-measured PA and ST and musculoskeletal health outcomes in middle-aged women.	ST was not associated with muscular strength and balance (*p* > 0.05).
21. Yasunaga et al. (2017) [56] Japan	287 (180 ♂; 107 ♀)	74.4 ± 5.2 (65–84 y)	Device: Active style Pro HJA-350IT; SB cut-point: ≤1.5 METs; Epoch: 60 s; Non-wear: 60 min; Minimum wear: ≥4 days (include 1 weekend day), ≥600 min/day; Average wear (days and min/day): 7.2 ± 0.9 days, 901.1 ± 87.5 min/day; Quantification of SB: Total ST (min/day).	Muscular strength (hand grip strength (kg)); balance (one-legged stance with eyes open (s)).	SB, PA, gait speed, balance, mobility, strength	To examine the associations of objectively-assessed ST and PA with performance-based physical function.	ST was not associated with muscular strength (r = −0.056, *p >* 0.05) and balance (r = −0.238, *p >* 0.05).

Note: *n*, subjects number; ♂, male; ♀, female; min/day, minutes per day; h/day, hours per day; CRF, cardiorespiratory fitness; PA, physical activity; ST, sedentary time; METs, Metabolic Equivalents; y, years; MVPA, moderate to vigorous physical activity; LPA, light physical activity; PAEE, physical activity energy expenditure; NA, not available; BMI, body max index; min, minutes; m, meters; reps, repetitions; s, seconds; cm, centimeters; kg, kilograms; m/s, meters per second.

## References

[1] Caspersen C.J., Powell K.E., Christenson G.M. (1985). Physical Activity, Exercise, and Physical Fitness: Definitions and Distinctions for Health-Related Research. Public Health Rep..

[2] Maslow A., Sui A., Lee D., Vuori I., Blair S. (2011). Fitness and Adiposity as Predictors of Functional Limitation in Adults. J. Phys. Act. Health.

[3] Rosengren B.E., Ribom E.L., Nilsson J.Å., Mallmin H., Ljunggren Ö., Ohlsson C., Mellström D., Lorentzon M., Stefanick M., Lapidus J. (2012). Inferior physical performance test results of 10.998 men in the MrOS Study is associated with high fracture risk. Age Ageing.

[4] Edholm P., Nilsson A., Kadi F. (2019). Physical function in older adults: Impacts of past and present physical activity behaviors. Scand. J. Med. Sci. Sports.

[5] McPhee J.S., French D.P., Jackson D., Nazroo J., Pendleton N., Degens H. (2016). Physical activity in older age: Perspectives for healthy ageing and frailty. Biogerontology.

[6] Kodama S., Saito K., Tanaka S., Maki M., Yachi Y., Asumi M., Sugawara A., Totsuka K., Shimano H., Ohashi Y. (2009). Aerobic endurance as a quantitative predictor of all-cause mortality and cardiovascular events in healthy men and women. A meta-analysis. JAMA.

[7] Leong D.P., Teo K.K., Rangarajan S., Lopez-Jaramillo P., Avezum A., Orlandini A., Seron P., Ahmed S.H., Rosengren A., Kelishadi R. (2015). Prognostic value of grip strength: Findings from the Prospective Urban Rural Epidemiology (PURE) study. Lancet.

[8] García-Hermoso A., Cavero-Redondo I., Ramírez-Vélez R., Ruiz J.R., Ortega F.B., Lee D.-C., Martínez-Vizcaíno V. (2018). Muscular Strength as a Predictor of All-Cause Mortality in an Apparently Healthy Population: A Systematic Review and Meta-Analysis of Data from Approximately 2 Million Men and Women. Arch. Phys. Med. Rehabil..

[9] Lin P.-S., Hsieh C.-C., Cheng H.-S., Tseng T.-J., Su S.-C. (2016). Association between Physical Fitness and Successful Aging in Taiwanese Older Adults. PLoS ONE.

[10] Moratalla-Cecilia N., Soriano-Maldonado A., Ruiz-Cabello P., Fernández M.M., Gregorio-Arenas E., Aranda P., Aparicio V.A. (2016). Association of physical fitness with health-related quality of life in early postmenopause. Qual. Life Res..

[11] Katzmarzyk P.T., Craig C.L. (2002). Musculoskeletal fitness and risk of mortality. Med. Sci. Sports Exerc..

[12] Breton É., Beloin F., Fortin C., Martin A., Ouellet M., Payette H., Levasseur M. (2014). Gender-specific associations between functional autonomy and physical capacities in independent older adults: Results from the NuAge study. Arch. Gerontol. Geriat..

[13] Davis M.G., Fox K.R., Stathi A., Trayers T., Thompson J.L., Cooper A.R. (2014). Objectively measured sedentary time and its association with physical function in older adults. J. Aging Phys. Act..

[14] Dogra S., Ashe M.C., Biddle S.J.H., Brown W.J., Buman M., Chastin S., Gardiner O., Inoue S., Jefferis B.J., Oka K. (2017). Sedentary time in older men and women: An international consensus statement and research priorities. Br. J. Sports Med. Publ..

[15] Panahi S., Tremblay A. (2018). Sedentariness and Health: Is Sedentary Behavior More Than Just Physical Inactivity?. Front. Public Health.

[16] Tremblay M.S., Aubert A., Barnes J.D., Saunders T.J., Carson V., Latimer-Cheung A.E., Chastin S.F.M., Altenburg T.M., Chinapaw M.J.M. (2017). Sedentary Behavior Research Network (SBRN)-Terminology Consensus Project process and outcome. Int. J. Behav. Nutr. Phys. Act..

[17] Loyen A., Clarke-Cornwell A.M., Anderssen S., Hagströmer M., Sardinha L.B., Sundquist K., Ekelund U., Steene-Johannessen J., Baptista F., Hansen B. (2017). Sedentary Time and Physical Activity Surveillance through Accelerometer Pooling in Four European Countries. Sports Med..

[18] Powell C., Herring M.P., Dowd K.P., Donnelly A.E., Carson B.P. (2018). The cross-sectional associations between objectively measured sedentary time and cardiometabolic health markers in adults—A systematic review with meta-analysis component. Obes. Rev..

[19] Ku P., Steptoe A., Liao Y., Hsueh M., Chen L. (2018). A cut-off of daily sedentary time and all-cause mortality in adults: A meta-regression analysis involving more than 1 million participants. BMC Med..

[20] Young D.R., Hivert M.F., Alhassan S., Camhi S.M., Ferguson J.F., Katzmarzyk P.T., Lewis C.E., Owen N., Perry C.K. (2016). Sedentary Behavior and Cardiovascular Morbidity and Mortality: A Science Advisory from the American Heart Association. Circulation.

[21] Cooper A.J.M., Simmons R.K., Kuh D., Brage S., Cooper R. (2015). Physical Activity, Sedentary Time and Physical Capability in Early Old Age: British Birth Cohort Study. PLoS ONE.

[22] Dogra S., Clarke J.M., Copeland J.L. (2017). Prolonged Sedentary time and physical fitness among Canadian men and women aged 60 to 69. Health Rep..

[23] Spartano N.L., Lyass A., Larson M.G., Trand T., Andersson C., Blease S.J., Esliger D.W., Vasan R.S., Murabito J.M. (2019). Objective physical activity and physical performance in middle-aged and older adults. Exp. Gerontol..

[24] Velde J.H.P.M., Savelberg H.H.C.M., van der Berg J.D., Sep S.J.S., van der Kallen C.J.H.M., Dagnelie P.C., Schram M.T., Henry R.M.A., Reijven P.L.M., van Geel T.A.C.M. (2017). Sedentary Behavior is Only Marginally Associated with Physical Function in Adults Aged 40–75 Years—The Maastricht Study. Front. Physiol..

[25] Dickie K., Micklesfield L.K., Chantler S., Lambert E.V., Goedecke J.H. (2015). Cardiorespiratory Fitness and Light-Intensity Physical Activity are Independently Associated with Reduced Cardiovascular Disease Risk in Urban Black South African Women: A Cross-Sectional Study. Metab. Syndr. Relat. Disord..

[26] Wientzek A., Tormo Díaz M.J., Castaño J.M.H., Amiano P., Arriola L., Overvad K., Østergaard J.N., Charles M.A., Fagherazzi G., Palli D. (2013). Cross-sectional associations of Objetively Measured Physical Activity, Cardiorrespiratory Fitness and Anthropometry in European Adults. Obesity.

[27] Edwards M.K., Loprinzi P.D. (2016). High Amounts of Sitting, Low Cardiorespiratory Fitness, and Low Physical Activity Levels: 3 Key Ingredients in the Recipe for Influencing Metabolic Syndrome Prevalence. Am. J. Health Promot..

[28] Velde J.H.P.M., Koster A., Berg J.D., Sep S.J.S., Kallen C.J.H., Dagnelie P.C., Schram M.T., Henry R.M.A., Eussen S.J.P., Van Dongen M.C.J.M. (2017). Sedentary Behavior, Physical Activity, and Fitness—The Maastricht Study. Med. Sci. Sports Exerc..

[29] Willoughby T., Copeland J.L. (2015). Sedentary time is not independently related to postural stability or leg strength in women 50–67 years old. Appl. Physiol. Nutr. Metab..

[30] Wu F., Laslett L.L., Oldenburg B., Jones G., Winzenberg T. (2017). Moderate-to-Vigorous Physical Activity but not Sedentary Time is Associated with Musculoskeletal Health Outcomes in a Cohort of Australian Middle-Aged Women. J. Bone Miner. Res..

[31] Foong Y.C., Chherawala N., Aitken D., Scott D., Winzenberg T., Jones G. (2016). Accelerometer-determined physical activity, muscle mass, and leg strength in community-dwelling older adults. J. Cachexia Sarcopenia Muscle.

[32] Reid N., Daly R.M., Winkler E.A.H., Gardiner P.A., Eakin E.G., Owen N., Dunstan D.W., Healy G.N. (2016). Associations of Monitor-Assessed Activity with Performance-Based Physical Function. PLoS ONE.

[33] Prioreschi A., Brage S., Westgate K., Norris S.A., Micklesfield L.K. (2017). Cardiorespiratory fitness levels and associations with physical activity and body composition in young South African adults from Soweto. BMC Public Health.

[34] Knaeps S., Lefevre J., Wijtzes A., Charlier R., Mertens E., Bourgois J.G. (2016). Independent Associations between Sedentary Time, Moderate-To-Vigorous Physical Activity, Cardiorespiratory Fitness and Cardio-Metabolic Health: A Cross-Sectional Study. PLoS ONE.

[35] Velde J.H.P.M.V., Savelberg H.H.C.M., Schaper N.C., Koster A. (2015). Moderate Activity and Fitness, Not Sedentary Time, Are Independently Associated with Cardio-Metabolic Risk in U.S. Adults Aged 18–49. Int. J. Environ. Res. Public Health.

[36] Atkin A.J., Gorely T., Clemes S.A., Yates T., Edwardson C., Brage S., Salmon J., Marshall S.J., Biddle S.J.H. (2012). Methods of Measurement in epidemiology: Sedentary Behaviour. Int. J. Epidemiol..

[37] Campbell S.D.I., Brosnan B.J., Chu A.K.Y., Skeaff C.M., Rehrer N.J., Perry T.L., Peddie M.C. (2018). Sedentary Behavior and Body Weight and Composition in Adults: A Systematic Review and Meta-analysis of Prospective Studies. Sports Med..

[38] Guthold R., Stevens G.A., Riley L.M., Bull F.C. (2018). Worldwide trends in insufficient physical activity from 2001 to 2016: A pooled analysis of 358 population-based surveys with 1·9 million participants. Lancet Glob. Health.

[39] Compernolle S., DeSmet A., Poppe L., Crombez G., Bourdeaudhuij I., Cardon G., Van der Ploeg H.P., Dyck D.V. (2019). Effectiveness of interventions using self-monitoring to reduce sedentary behavior in adults: A systematic review and meta-analysis. Int. J. Behav. Nutr. Phys. Act..

[40] Moher D., Liberati A., Tetzlaff J., Altman D.G., The PRISMA Group (2009). Preferred Reporting Items for Systematic Reviews and Meta-Analyses: The PRISMA Statement. PLoS Med..

[41] World Health Organization (2010). Global Recommendations on Physical Activity for Health.

[42] Panic N., Leoncini E., de Belvis G., Ricciardi W., Boccia S. (2013). Evaluation of the endorsement of the preferred reporting items for systematic reviews and meta-analysis (PRISMA) statement on the quality of published systematic review and meta-analyses. PLoS ONE.

[43] Malta M., Cardoso L.O., Bastos F.I., Magnanini M.M.F., Silva C.M. (2010). Iniciativa STROBE: Subsídios para a comunicação de estudos observacionais. Rev. Saúde Pública.

[44] von Elm E., Altman D.G., Egger M., Pocock S.J., Gøtzsche P.C., Vandenbroucke J.P. (2007). The strengthening the reporting of observational studies in epidemiology (STROBE) statement: Guidelines for reporting observational studies. Lancet.

[45] Sterne J.A.C., Sutton A.J., Loannidis J.P.A., Terrin N., Jones D.R., Lau J., Carpenter J., Rücker G., Harboard R.M., Scmid C.H. (2011). Recommendations for examining and interpreting funnel plot asymmetry in meta-analyses of randomized controlled trials. BMJ.

[46] Borenstein M., Hedges L.V., Higgins J.P.T., Rothstein H.R. (2010). A basic introduction to fixed-effect and random-effects models for meta-analysis. Res. Synth. Methods.

[47] Higgins J.P.T., Thompson S.G., Deeks J.J., Altman D.G. (2003). Measuring inconsistency in meta-analyses. BMJ.

[48] Egger M., Smith G.D., Schneider M., Minder C. (1997). Bias in meta-analysis detected by a simple, graphical test. BMJ.

[49] Burzynska A.Z., Chaddock-Heyman L., Voss M.W., Wong C.N., Gothe N.P., Olson E.A., Knecht A., Lewis A., Monti J.M., Cooke G.E. (2014). Physical Activity and Cardiorespiratory Fitness are Beneficial for White Matter in Low-Fit Older Adults. PLoS ONE.

[50] Jantunen H., Wasenius N., Salonen M., Perala M., Osmond C., Kautianen H., Simonen M., Pohjolainen P., Kajantie E., Rantanen T. (2017). Objectively measured physical activity and physical performance in old age. Age Ageing.

[51] Gennuso K.P., Thraen-Borowski K.M., Gangnon R.E., Colbert L.H. (2015). Patterns of sedentary behaviour and physical function in older adults. Aging Clin. Exp. Res..

[52] Liao Y., Hsu H., Ishii K., Koohsari M.J., Oka K. (2018). Associations of total amount and patterns of objectively measured sedentary behaviour with performance-based physical function. Prev. Med. Rep..

[53] Silva F.M., Petrica J., Serrano J., Paulo R., Ramalho A., Lucas D., Ferreira J.P., Duarte-Mendes P. (2019). The Sedentary Time and Physical Activity Levels on Physical Fitness in the Elderly: A Comparative Cross-Sectional Study. Int. J. Environ. Res. Public Health.

[54] Santos D., Silva A., Baptista F., Santos R., Vale S., Mota J., Sardinha L. (2012). Sedentary behavior and physical activity are independently related to functional fitness in older adults. Exp. Gerontol..

[55] Savikangas T., Tirkkonen A., Alen M., Rantanen T., Fielding R.A., Rantalainen T., Sipilä S. (2020). Associations of physical activity in detailed intensity ranges with body composition and physical function. A cross-sectional study among sedentary older adults. Eur. Rev. Aging Phys. Act..

[56] Yasunaga A., Shibata A., Ishii K., Koohsari M.J., Inoue S., Sugiyama T., Owen N., Oka K. (2017). Associations of sedentary behavior and physical activity with older adults’ physical function: An isotemporal substitution approach. BMC Geriatr..

[57] Zeiher J., Ombrellaro K.J., Perumal N., Keil T., Mensink G.B.M., Finger J.D. (2019). Correlates and Determinants of Cardiorespiratory Fitness in Adults: A Systematic Review. Sports Med..

[58] Cliff D.P., Hesketh K.D., Vella S.A., Hinkley T., Tsiros M.D., Ridgers N.D., Carver A., Veitch J., Parrish A.-M., Hardy L.L. (2016). Objectively measured sedentary behaviour and health and development in children and adolescents: Systematic review and meta-analysis. Obes. Rev..

[59] Thijssen D.H., Green D.J., Hopman M.T. (2011). Blood vessel remodeling and physical inactivity in humans. J. Appl. Physiol..

[60] Wang D.X.M., Yao J., Zirek Y., Reijnierse E.M., Maier A.B. (2020). Muscle mass, strength, and physical performance predicting activities of daily living: A meta-analysis. J. Cachexia Sarcopenia Muscle.

[61] Wullems J.A., Verschueren S.M.P., Degens H., Morse C., Onambélé G.L. (2016). A review of the assessment and prevalence of sedentarism in older adults, its physiology/health impact and non-exercise mobility counter-measures. Biogerontology.

[62] Chastin S.F.M., Ferriolli E., Stephens N.A., Fearon K.C.H., Greig C. (2012). Relationship between sedentary behaviour, physical activity, muscle quality and body composition in healthy older adults. Age Ageing.

[63] Gianoudis J., Bailey C.A., Daly R.M. (2015). Associations between sedentary behaviour and body composition, muscle function and sarcopenia in community-dwelling older adults. Osteoporos. Int..

[64] Westbury L.D., Fuggle N.R., Syddall H.E., Duggal N.A., Shaw S.C., Maslin K., Dennison E.M., Lord J.M., Cooper C. (2018). Relationships between Markers of Inflammation and Muscle Mass, Strength and Function: Findings from the Hertfordshire Cohort Study. Calcif. Tissue Int..

[65] Schaap L.A., Pluijm S.M.F., Deeg D.J.H., Harris T.B., Kritchevsky S.B., Newman A.B., Colbert L.H., Pahor M., Rubin S.M., Tylavsky F.A. (2009). Higher Inflammatory Marker Levels in Older Persons: Associations with 5-Year Change in Muscle Mass and Muscle Strength. J. Gerontol. A Biol. Sci. Med. Sci..

[66] Lord S.R., Clark R.D., Webster I.W. (1991). Postural Stability and Associated Physiological Factors in a Population of Aged Persons. J. Gerontol. A Biol. Sci. Med. Sci..

[67] Choy N.L., Brauer S., Nitz J. (2003). Changes in postural stability in women aged 20 to 80 years. J. Gerontol. A Biol. Sci. Med. Sci..

[68] Medeiros H.B., de Araujo D.S., de Araujo C.G. (2013). Age-related mobility loss is joint-specifc: An analysis from 6000 Flexitest results. Age.

[69] Zhao Y., Chung P. (2016). Differences in function fitness among older adults with and without risk of falling. Asian Nurs. Res..

[70] Mañas A., Del Pozo-Cruz B., García-García F.J., Guadalupe-Grau A., Ara I. (2017). Role of objectively measured sedentary behaviour in physical performance, frailty and mortality among older adults: A short systematic review. Eur. J. Sport Sci..

[71] Wirth K., Klenk J., Brefka S., Dallmeier D., Faehling K., Figuls M.R., Tully M.A., Giné-Garriga M., Caserotti P., Salvà A. (2017). Biomarkers associated with sedentary behaviour in older adults: A Systematic review. Ageing Res. Rev..

[72] Biddle S.J., Bennie J.A., De Cocker K., Dunstan D., Gardiner P.A., Healy G.N., Lynch B., Owen N., Brakenridge C., Brown W. (2019). Controversies in the Science of Sedentary Behaviour and Health: Insights, Perspectives and Future Directions from the 2018 Queensland Sedentary Behaviour Think Tank. Int. J. Environ. Res. Public Health.

[73] Lavie C.J., Ozemek C., Carbone S., Katzmarzyk P.T., Blair S.N. (2019). Sedentary Behavior, Exercise, and Cardiovascular Health. Circ. Res..

[74] Thyfault J.P., Du M., Kraus W.E., Levine J.A., Booth F.W. (2015). Physiology of Sedentary Behavior and its Relationship to Health Outcomes. Med. Sci. Sports Exerc..

[75] Migueles J.H., Cadenas-Sanchez C., Ekelund U., Nyström C.D., Mora-Gonzalez J., Löf M., Labayen I., Ruiz J.R., Ortega F.B. (2017). Accelerometer data collection and processing criteria to assess physical activity and other outcomes: A systematic review and practical considerations. Sports Med..

